# The use of simplified verbal autopsy in identifying causes of adult death in a predominantly rural population in Ethiopia

**DOI:** 10.1186/1471-2458-5-58

**Published:** 2005-06-03

**Authors:** Kidest Lulu, Yemane Berhane

**Affiliations:** 1 Department of Community Health, Jimma University, Jimma, Ethiopia; 2 Department of Community Health, Faculty of Medicine, Addis Ababa University, P.o. Box 2077, Addis Ababa, Ethiopia

## Abstract

**Background:**

Information on adult mortality is essentially non-existent in Ethiopia particularly from rural areas where access to health services is limited and most deaths occur at home. This study was conducted with the aim of identifying causes of adult death in a rural population of Ethiopia using a simplified verbal autopsy instrument.

**Methods:**

All deaths in the age-group 15–49 years during the period of 1995–99 were taken from computerized demographic surveillance database maintained by the Butajira Rural Health Program. Data on the causes of death were collected from close relatives of the deceased persons by lay interviewers. Causes of death were diagnosed using "expert algorithm" programmed onto a computer.

**Results:**

The major causes of death were acute febrile illnesses (25.2%), liver diseases (11.3%), diarrheal diseases (11.1%), tuberculosis (9.7%) and HIV/AIDS (7.4%). Overall communicable diseases accounted for 60.8% of the deaths. The high levels of mortality from communicable diseases reflect the poor socioeconomic development of the country, and the general poor coverage of health and education services in rural Ethiopia. The tools used in this study can easily be added-on to the numerous health surveys conducted in the country.

**Conclusion:**

The simplified approach to verbal autopsy diagnosis can produce useful data that can effectively guide priority health interventions in rural areas where routine information system is either very weak or non-existent.

## Background

Mortality statistics have several advantages when compared to other sources of health information such as morbidity data as death is a unique event that is remembered for long time and because of its finality it is clearly defined. Thus, statistics on cause of death are reliable and useful in guiding priority interventions in public health [1].

In developing countries, where most deaths are neither attended by doctors nor medically certified, information on causes of death is usually incomplete and of poor quality [2]. To alleviate the problem in resource poor countries an indirect method called verbal autopsy (VA), which makes use of lay reporters, has been adopted to identify causes of death. It uses information on the circumstances leading to death, symptoms and signs during the terminal illness, obtained from the family of the deceased, to assign cause of death. VA data are generated through retrospective questioning in surveys or in demographic surveillance systems [3-6].

The VA technique is based on the assumption that most causes of death have distinct symptom complexes that can be recognized, remembered and reported by lay respondents. It assumes that it is possible to classify deaths based on the reported information into useful categories of causes of death. The validity of VA is influenced by the type of illness leading to death, characteristics of the deceased person, and other factors related to the classification of causes of death, as well as the design and content of the questionnaire and field procedures [5,7,8].

Factors that affect the validity of a VA include the approach to mortality classification, the design of the questionnaire, the methods of deriving a diagnosis, and the number and combinations of categories of causes of death assigned. The choice of categories of causes of death determines the complexity of diagnostic algorithms. A classification approach using fewer categories will group causes of death with closely related symptom complexes together and hence tends to increase the validity of the VA but provides less detailed information. The other advantage in broadly classifying mortality is that the classification can be used in a wide range of settings with minor modifications [5].

The type of questionnaire also influences the validity of the VA outcome. An open format VA questionnaire would require skilled and possibly medically trained interviewers, and would also have a higher inter-interviewer variability. A checklist with filters on the other hand would not need medically trained interviewers, and would be more efficient for data collection. But, a false negative answer to a filter question will result in omitting one whole disease category and hence decrease the sensitivity of the VA. A checklist without filters would not require a medically trained person and would reduce interviewer bias, but it may not capture all details of the symptoms leading to death and may increase the number of falsely reported symptoms [5].

Different validation studies for the VA method have been done for deaths in children [9-12], and adults [6,13] suggesting that the VA method is an important tool for diagnosing causes of death, especially in developing countries. Studies in Tanzania [14] and in Ethiopia [15] have shown the robustness of VA method in identifying cause of death using lay interviewers.

Trends in cause-specific mortality over time, differentials of mortality between different population groups, or evaluation of the effect of interventions can be studied from data using the VA method. Additionally, they can be used to establish the relative public health importance of different causes of death in the populations in order to identify priorities and appropriate interventions [5,16]

Ethiopia is a country where low standards of living, poor environmental conditions and under developed social services have caused serious impact on the health status of the population [17,18]. Although adults are the pillar of the society as economically productive, biologically reproductive, and responsible for the support of children and elderly dependents their health needs receive little attention in developing countries [19]. Information on adult health conditions is essentially non-existent in most developing countries as the routine system does not cover large segment of the population, especially those living in the rural areas [2,20,21]. In Ethiopia, due to the absence of vital event registration system and inaccessibility of health facilities most deaths occur at home and pass undocumented. This study was done with the objective of identifying the causes of death in a predominantly rural population where almost all deaths are occurring at home. The paper tries to demonstrate a possibility of using a relatively simple and inexpensive verbal autopsy method for obtaining valuable data in improving health services to the rural underserved populations.

## Methods

The study district, Meskan and Mareko, is located in the central-south part of Ethiopia at an average altitude of 2,100 meters above sea level with a range of 1,750 m in the low lands to 3,400 m in the mountainous areas. The actual study sites were the nine rural and one urban study *kebeles *(*Kebele *is the smallest administrative unit in Ethiopia often having an average of 500 households, the size varies significantly from district to district) that are under continuous demographic surveillance by the Butajira Rural Health Program (BRHP). These sites were randomly selected using the probability proportionate to size technique. The BRHP registers events such as birth, death, marriage, new household, out-migration, in-migration, and internal move. Information on the circumstances leading to death was not routinely collected by the surveillance system. Data on mortality as well as the above mentioned events were collected monthly, by trained village-based enumerators, through household visits. The total population of the area was estimated to be 257,000 in 1999. Detailed description of the study area is given elsewhere [22].

All adult deaths in the age group 15 to 49 year that were registered by the BRHP from January 1995 to December 1999 were identified from the longitudinal surveillance database and considered for this study. The age-group 15–49 was selected for this study in order to identify the causes of premature adult death, since life expectancy at birth in Ethiopia is 53 years for females and 50.9 years for males [17,23].

Data were collected using a simplified verbal autopsy questionnaire administered to relatives of the deceased. The questionnaire for this study was developed based on the WHO questionnaire for identifying cause of death in children and modified to address adult signs and symptoms of diseases [8]. It was initially prepared in English and then translated to Amharic (the national language). The first part of the questionnaire has questions on the socio-demographic characteristics of the subjects such as area of residence, age, sex, marital status, education, economic status, health service usage; and their personal habits such as smoking and alcohol drinking. The second part has questions on circumstances surrounding mortality such as signs and symptoms of illness, duration of illness, health care seeking, medications used, and admission to a health facility. The interview on average took about 20 minutes. The VA questionnaire used in this study is short and simplified. That was done for the following reasons: to develop a less complicated questionnaire that can be used by lay interviewers with minimal training; to get adequate response to all questions from rural and illiterate people; to make the questionnaire suitable for annexing into routine surveillance systems in the future; to facilitate the development of an uncomplicated algorithm for identifying broad categories of cause of death; and to obtain fairly complete information about each death. Data collectors and supervisors were recruited from the study area. The data collectors were all high school graduates with good experience in a similar type of fieldwork. Data collectors went house to house to collect information about the deceased person identified from the study base. In the event respondents were not available on first visit, repeated visits were arranged on at least two occasions.

Data entry and analysis were done using Epi Info version 6.04 statistical package. Causes of death were analyzed using an expert algorithm programmed into a computer. The algorithm does not include cancer as one category mainly because health records did not show cancer as major causes of either hospital admission or hospital death for adults in Ethiopia [17,23]. The second reason is that because of the varied manifestations for different types of cancers, it would be difficult to accommodate deaths due to cancer into the simple questionnaire and algorithm used in this study. The algorithms used are shown in Table 1.

**Table 1:** Algorithm used for the diagnoses of cause of death, in Meskan and Mareko district, Ethiopia. 2000.

**Algorithm**	**Diagnosis**
Duration of illness < 30 days + Accidents (intentional or unintentional)	Injuries
Female sex + (Pregnant or In labor or in the puerperal period)	Maternal causes
Duration of illness > 30 days + Cough + Weight loss + (Bloody sputum or Fever or Ascites) + no diarrhea	Tuberculosis
Duration of illness > 30 days + Cough + Diarrhea + Fever + Weight loss	AIDS
Duration of illness > 15 days + (Edema of legs or Ascites) + Jaundice	Liver diseases
Duration of illness < 15 days + Abdominal swelling + Repeated vomiting + No diarrhea	Acute abdomen
Duration of illness > 30 days + cough + Dyspnea + Wheezing + No bloody sputum	Chronic obstructive lung diseases
Duration of illness < 30 days + Diarrhea + No cough	Diarrheal diseases
Duration of illness < 15 days + Fever + Headache	Acute febrile illness
Duration of illness < 15 days + Fever + Headache + Neck stiffness	Meningitis
Duration of illness < 30 days + Cough + Fever + (Dyspnea or Chest pain)	Pneumonia
Duration of illness < 30 days + Dyspnea + Palpitation + (Edema of legs or Ascites)	Cardio Vascular diseases

## Results

A total of 819 adult (15–49 years) deaths were identified from the BRHP computer database for the period 1995–1999. In the field appropriate respondents (family members and close relatives) were identified for 515 (63%) of the deceased persons. The reasons for not identifying the rest include out-migration of the entire family of the deceased person, unavailability of relatives on repeated visits, wrong recorded information about the deceased, and double recording. The highest proportion of the missed cases (59.7%) was for those who died in the initial one and half years of the study. From those who died in the last one and half years of the study only 19.4% of the cases were missed. Communicable diseases accounted for 60.8% of the deaths, non-communicable for 24.1%, maternal causes 7%, injuries 1.7%, and undetermined causes consisted of 6.4%. The highest number of deaths was due to acute febrile illnesses (AFI) comprising 25.2% of all causes. AFI, diarrheal diseases, tuberculosis, AIDS, and liver diseases together accounted for 64.7% of the deaths (Table 1). AFI was the cause for 28% and 25.5% of the deaths in the rural highland, and lowland areas respectively, while it accounted to only 15.5% of the urban deaths. Diarrheal diseases accounted for 7% of the deaths in the urban area, 10.6% in the rural highland and 13% in the rural lowland. However, HIV/AIDS in the urban area accounted for 11.3% in contrast to 8.5% and 4.8% in the rural highland and lowland (Table 2).

**Table 2:** Possible verbal autopsy based causes of death by area of residence in Meskan and Mareko district, Ethiopia. 1995–1999.

Possible cause	Urban	Rural highland	Rural lowland	Total
	No. (%)	No. (%)	No. (%)	No. (%)
I. Communicable Diseases	37 (52.1)	150 (63.6)	126 (60.6)	313 (60.8)
Acute Febrile illnesses	11 (15.5)	66 (28.0)	53 (25.5)	130 (25.2)
Diarrheal diseases	5 (7.0)	25 (10.6)	27 (13.0)	57 (11.1)
All forms of tuberculosis	6 (8.5)	23 (9.7)	21 (10.1)	50 (9.7)
HIV/ AIDS	8 (11.3)	20 (8.5)	10 (4.8)	38 (7.4)
Pneumonia	4 (5.6)	14 (5.9)	10 (4.8)	28 (5.4)
Meningitis	3 (4.2)	2 (0.8)	5 (2.4)	10 (1.9)
				
II. Non-communicable Diseases	20 (28.2)	56 (23.7)	48 (23.1)	124 (25.4)
Liver diseases	8 (11.3)	26 (11.1)	24 (11.5)	58 (11.3)
Cardio-vascular diseases	6 (8.5)	13 (5.5)	13 (6.3)	32 (6.2)
Chronic obstructive lung diseases	4 (5.6)	14 (5.9)	9 (4.3)	27 (5.2)
Acute abdomen conditions	2 (2.8)	3 (1.3)	2 (0.9)	7 (1.4)
				
III. Maternal causes	3 (4.2)	19 (8.1)	14 (6.7)	36 (7.0)
				
IV. Injuries	2 (2.8)	6 (2.5)	1 (0.5)	9 (1.7)
				
Undetermined	9 (12.7)	5 (2.1)	19 (9.1)	33 (6.4)
Total	71 (100)	236 (100)	208 (100)	515 (100.0)

In general, deaths from communicable diseases was greater in males (62.8%) than in females (58.7%); while deaths from non-communicable diseases was greater in females (35.4%) than in males (26.8%). Maternal mortality accounted for 7% of all deaths, and 14.2% of the female deaths (Table 3). Of the 36 women identified to have died from maternal causes; 17 (47.2%) had fever, 10 (27.8%) had prolonged labor, and 4 (11.1%) had hemorrhage.

**Table 3:** Possible verbal autopsy causes of death by sex in Meskan and Mareko district, Ethiopia. 1995–1999.

Possible cause	Females	Males	Total
	Number (%)	Number (%)	Number (%)
I. Communicable Diseases	149 (58.7)	164 (62.8)	313 (60.8)
Acute Febrile illnesses	63 (24.8)	67 (25.7)	130 (25.2)
Diarrheal diseases	28 (11.0)	29 (11.1)	57 (11.1)
All forms of tuberculosis	21 (8.3)	29 (11.1)	50 (9.7)
HIV/ AIDS	19 (7.5)	19 (7.3)	38 (7.4)
Pneumonia	17 (6.7)	11 (4.2)	28 (5.4)
Meningitis	1 (0.4)	9 (3.4)	10 (1.9)
			
II. Non-communicable Diseases	54 (21.2)	78 (26.8)	124 (25.4)
Liver diseases	26 (10.2)	32 (12.3)	58 (11.3)
Cardio-vascular diseases	14 (5.5)	18 (6.9)	32 (6.2)
Chronic obstructive lung diseases	10 (3.9)	17 (6.5)	27 (5.2)
Acute abdomen conditions	4 (1.6)	3 (1.1)	7 (1.4)
			
III. Maternal causes	36 (14.2)	---	36 (7.0)
			
IV. Injuries	3 (1.2)	6 (2.3)	9 (1.7)
			
Undetermined	12 (4.7)	21 (8.1)	33 (6.4)
Total	254 (100.0)	261(100.0)	515(100.0)

Since a large proportion of the deaths that occurred in the first one and half year of the study were missed, analysis of cause of death was also made for the deaths excluding the initial one and half year. There were a total of 422 cases found in this category. Communicable diseases accounted for 59.2% of the deaths, non-communicable 25.4%, maternal causes 6.9%, injuries 0.9% and undetermined causes were 7.6% (Table 4).

**Table 4:** Possible verbal autopsy based causes of death for the last three and half years of the recall period as compared to the total five years in Meskan and Mareko district, Ethiopia. 1995–1999.

Possible cause	Year of death	
	
	09/01/96-12/31/99	01/01/95-12/31/99
	Number (%)	Number (%)
I. Communicable Diseases	250 (59.2)	313 (60.8)
Acute Febrile illnesses	102 (24.2)	130 (25.2)
Diarrheal diseases	47 (11.1)	57 (11.1)
All forms of tuberculosis	42 (10.0)	50 (9.7)
HIV/ AIDS	30 (7.1)	38 (7.4)
Pneumonia	22 (5.2)	28 (5.4)
Meningitis	7 (1.7)	10 (1.9)
		
II. Non-communicable Diseases	95 (25.4)	124 (25.4)
Liver diseases	49 (11.6)	58 (11.3)
Cardio-vascular diseases	29 (6.9)	32 (6.2)
Chronic obstructive lung diseases	23 (5.5)	27 (5.2)
Acute abdomen conditions	6 (1.4)	7 (1.4)
		
III. Maternal causes	29 (6.9)	36 (7.0)
		
IV. Injuries	4 (0.9)	9 (1.7)
		
Undetermined	32 (7.6)	33 (6.4)
Total	422 (100.0)	515 (100.0)

## Discussion

This study has attempted to identify the causes of death at a district level based on a simplified verbal autopsy procedure. Although information of this type is recognized to be very important for health planning and decision making it is largely nonexistent in developing countries, especially in rural settings. In the study area the major causes of death were acute febrile illnesses, liver diseases, diarrheal diseases, tuberculosis and HIV/AIDS. Maternity related causes accounted for 14.2% of the total female deaths. The questionnaire was made short and easily understandable to enhance its application on illiterate rural respondents by lay interviewers. The simplification has narrowed the possibilities of making very specific diagnoses that are claimed to be achieved using a more elaborate questionnaire. On the other hand, it would have been impossible to obtain complete data using elaborated questionnaire administered by lay interviewers, especially for the deaths that occurred long ago. In rural areas it is very difficult, if not impossible, to find interviewers with fairly high level of education that can administer and record an elaborate open ended questionnaire. In this study no major problem was detected on the completed questionnaires that can be attributed to the data collector's incompetence.

Information was collected as far back as five years. We observed that the loss of the beloved is unforgettable in the study community and reported without any reservation, especially for adults. The recall period however can affect the quality of detailed information collected for VA use. Baqui utilized a five year recall period for children in Bangladesh and reported that increase recall lapse has no clear adverse effect on data quality [24]. In addition, the questions on events surrounding mortality in this study are very simple and direct. For example, one of the questions was on duration of illness in general, not on duration of specific symptoms. The omission of very specific questions was helpful in minimizing the problem of recall. The major problem associated with the long time span was the unavailability of relatives of the deceased for interview, due to out migration or other reasons. There was a marked decline in the proportion of the missed cases from 1995 to 1999. The high number of missed cases for the deaths during "01/01/95"-"08/31/97" may have affected the results of this study and can be sighted as one of the limitations encountered. This indicates that VA information need to be gathered closer to the event, i.e. within a time span of not more than two years. The simplified algorithm used for diagnosis of cause of death classified illnesses into "broad" categories consisting of 12 causes of death. For example all acute febrile illnesses and all forms of tuberculosis were lumped into one category. Although this "broad" classification has the disadvantage of diagnosing only a limited variety of diseases and leaving out some diseases as undetermined, it makes easier the use of VA and the algorithm in many similar settings once validated. Other studies have demonstrated that simplicity of the questionnaire, the use of lay interviewers, lack of complexity in the algorithm used, and the broad categories of diseases increase the validity of a study and its reproducibility in other settings [6].

Although a validation study was not done on the "expert algorithm" used, the combinations of symptoms and duration of illness used for diagnosing injuries, maternal deaths, acute febrile illnesses, and meningitis are believed to be highly sensitive but not very specific. For example the algorithm of AFI has the combination of "duration of illness less than 15 days", "fever" and "headache". This combination can pick almost all deaths due to AFIs, but it increases the false positivist.

The algorithm used for tuberculosis, AIDS, liver diseases, and pneumonia are less sensitive but more specific than the previously stated diseases categories, because of the sign and symptom combinations used. A validation study for verbal autopsy in adults had shown that the cause specific mortality fraction obtained using "expert algorithms" was within ± 20% of the gold standard for malaria, meningitis, tuberculosis/AIDS, acute abdomen conditions, diarrheal diseases, direct maternal causes, chronic liver diseases and renal disorders [14].

The algorithm used for cardiovascular diseases and chronic obstructive pulmonary diseases may not have performed well because of the unstable nature of the diseases resulting in difficulty of sighting symptoms specific to them [7]. The "expert algorithm" used in this study may not have given the correct diagnosis for some of the diseases at the individual level. However, in the face of absence of information on causes of mortality in Ethiopia, the information generated at the population level using these combinations is very useful in identifying priority health problems that need urgent intervention in rural populations.

Causes of death diagnosed through the expert algorithm showed that one fourth of the deaths were due to acute febrile illnesses. The proportion was much higher in the rural areas than in the town. In contrast, HIV/AIDS accounted for a higher proportion in the town than the rural areas. A previous study in the same area has shown a similar pattern of disease occurrence [16].

We have tried to see if there is any inconsistency in the causes of death as a result of the greater number of missed cases in the first one and half year of the study period. The deaths during the later period of the study have very similar pattern when compared to the total five year deaths. For communicable diseases it was 60.8% vs. 59.2%, for non-communicable 24.1% vs. 25.4% and for maternal causes 7% vs. 6.9%. This consistency can be taken as an indicator of non-differential loss of cases over the specified period of time. This also indicate the robustness of the approach even when data are not complete, which is truly the case in most rural settings of developing countries.

This study has grossly indicated the magnitude of female deaths due to maternal causes, which is very difficult to come by as measuring maternal mortality ratio is very expensive [25-27], such information could be helpful to implement practical interventions locally. Considerations in the line of using the probabilistic approach to interpreting verbal autopsies described by Byass might help to further simplify the methodology and facilitate its application in large surveys [28]. However, it is important to note that the questionnaire in this study was tailored to yield maximum information from mostly illiterate and rural people as administered by lay interviewers. Hence its applicability might be limited to situations similar to the study area both in terms of availability of information and profile of health problems, which is dominated by communicable diseases.

## Conclusion

In conclusion, this study provides evidence that interviewing deceased relatives using a simplified VA questionnaire and expert algorithm for diagnosis of causes of death can provide information on priority health problems in resource poor communities of developing countries. The importance of having local information for planning appropriate health interventions at district level is demonstrated in the same area [29]. We encourage more researches to further develop this simplified VA questionnaire and expert algorithm, and to do a validation study in order to enhance its applicability in larger scale health surveys.

## Competing interests

The author(s) declare that they have no competing interests.

## Authors' contributions

KL participated in the design of the study, field data collection, data analysis, and prepared the draft manuscript. YB participated in the design of the study, field data collection, and data analysis and revised the draft manuscript. Both authors read and approved the final manuscript.

## Pre-publication history

The pre-publication history for this paper can be accessed here:


